# Progression of diet induced nonalcoholic steatohepatitis is accompanied by increased expression of kruppel-like-factor 10 in mice

**DOI:** 10.1186/1479-5876-12-186

**Published:** 2014-07-01

**Authors:** Ja Kyung Kim, Kwan Sik Lee, Hye Young Chang, Woon Kyu Lee, Jung Il Lee

**Affiliations:** 1 Department of Internal Medicine, Gangnam Severance Hospital, Yonsei University College of Medicine, 211 Eunju-ro, Gangnam-gu, Seoul 135-720, Republic of Korea; 2 Medical Research Center, Gangnam Severance Hospital, 211 Eunju-ro, Gangnam-gu, Seoul 120-752, Republic of Korea; 3 Laboratory of Developmental Genetics, Inha University School of Medicine, Incheon 400-712, Republic of Korea

## Abstract

**Background:**

Kruppel-like-factor (KLF) 10 is identified as transforming growth factor (TGF) β inducible early gene and is reported to suppress lipogenic genes. Although previous studies report that TGFβ plays an important role in progression of nonalcoholic steatohepatitis (NASH) by regulating liver fibrosis, the association of KLF10 and NASH has never been explored. Thus we evaluated expressions and changes of *KLF10* in diet induced NASH and in NASH which was alleviated by ursodeoxycholic acid (UDCA). We also assessed *KLF10* in quiescent and activated hepatic stellate cells (HSCs).

**Methods:**

C57BL/6 mice were given high fat, sucrose diet (HFSD) at least for 12 weeks up to 48 weeks and sacrificed at 12, 24 and 48 weeks thereafter. In other groups, either standard diet (SD) or HFSD was given for 24 weeks at which point mice fed with HFSD were divided into two groups, and were given either UDCA in combination with HFSD or vehicle with HFSD. Mice under SD were given vehicle. HSCs were isolated from C57BL/6 mice in order to evaluated KLF10 expression in activated HSCs.

**Results:**

The mice were found to acquire liver steatosis and inflammation starting from week 12 of HFSD feeding, although significant liver fibrosis was noticed by week 24. Increased *TGFβ* and *collagen α1(I)* (*Col1α(I)*) expression was also apparent from week 24. However, expression of *KLF10* mRNA started to increase from week 12, earlier than *TGFβ* gene. Up-regulation of *KLF10* was accompanied by suppressed carbohydrate response element-binding protein (ChREBP) that is known to be protective against insulin resistance. The mice fed with HFSD and UDCA had decreased *Colα(I)* mRNA that was coincided with reduced *TGFβ* and *KLF10* expression. Expression of *ChREBP* was also recovered by UDCA administration. Enhanced *KLF10* was noticed in activated HSCs when quiescent cell showed minimal expression.

**Conclusions:**

Our study demonstrated that *KLF10* expression was significantly increased in diet induced NASH and collagen producing activated HSCs. We also noticed that this up-regulation of *KLF10* was accompanied by increased TGFβ signaling genes and suppressed *ChREBP* expression. These observations suggest possible association of KLF10 and NASH progression.

## Introduction

Nonalcoholic fatty liver disease (NAFLD) is the most prevalent chronic liver disease worldwide, ranging from simple steatosis to steatohepatitis [1-3]. Unlike simple steatosis, nonalcoholic steatohepatitis (NASH) is characterized by hepatocellular injury with inflammation and fibrosis that can progress to liver cirrhosis [4]. The presence of fibrosis and its severity is reported to be the most important prognostic factor [5-7].

Studies on the intriguing mechanism of liver fibrosis identify transforming growth factor (TGF) β as a key regulator of fibrosis [8-11]. However, being a multifunctional cytokine which regulates cell survival, differentiation, migration as well as extracellular matrix production [12,13], targeting TGFβ itself in treating liver fibrosis may accompany detrimental adverse effects. Studies on the signaling of TGFβ reveal that once it binds to a receptor which is then phosphorylated, subsequent intracellular signaling pathway including Smad proteins would activate [14]. In addition, TGFβ can induce immediate early response transcription factors such as Kruppel-like-factors (KLF) that would work as effector proteins [15,16].

KLF10, which is initially identified from osteoblastic cell population [17], is known to be regulated by TGFβ/Smad pathway. KLF10 may induce transcription of Smad2 and repress Smad7 [18-20]. It exerts its function as modulating differentiation markers, suppressing proliferation and inducing apoptosis of cells [18,21,22]. A recent study reports that KLF10 can also suppress lipogenic genes and that glucose stimulation induces *KLF10* mRNA expression, suggesting the possible role of KLF10 on glucose and lipid metabolism [23], two important pathways in relation to nonalcoholic fatty liver disease.

Regarding glucose and lipid metabolism, carbohydrate response element-binding protein (ChREBP) is known to induce the expression of lipogenic and glycolytic genes in response to the glucose stimulation [24,25]. Interestingly, overexpression of ChREBP in mice fed with high fat diet showed improved insulin resistance despite the greater hepatic steatosis [26]. On the other hands, ChREBP overexpression induces *KLF10* mRNA expression in rat hepatocytes whereas KLF10 overexpression partially suppressed ChREBP target genes [27].

KLF10 and its association with NASH have never been studied. Here, we used diet which was high in fat and sucrose to induce NASH and evaluated the expression of *KLF10* in the liver. We also evaluated the change of *KLF10* when diet induced NASH was alleviated after giving ursodeoxycholic acid (UDCA). Since our observations suggested that *KLF10* expression increased with progression of NASH fibrosis and repressed as fibrosis regressed, in vitro study using primarily cultured hepatic stellate cells (HSCs), key players of liver fibrosis, was done. Extracellular matrix (ECM) producing activated HSCs showed increased *KLF10* expression when compared with that of quiescent HSCs. We additionally identified that expression of *ChREBP* was suppressed in NASH and *KLF10* expression was inversely coincided with *ChREBP* in the liver.

## Materials and methods

### Animals and experimental design

The animal experimental procedures and protocols were approved by the Institutional Animal Care and Use Committee (IACUC) of Gangnam Severance Hospital, Yonsei University College of Medicine (Permit Number: 0173). The study was carried out in accordance with the recommendation and restrictions of IACUC.

C57BL/6 male mice (8 weeks of age) were obtained from the Central Lab Animal (Seoul, Korea) and housed with a 12-h light-dark cycle. Zeitgeber time zero (ZT0) referred to the time of lights on. Mice were fed with either a standard chow (standard diet, SD) or a diet high in saturated fat, cholesterol and sucrose, termed as high fat and sucrose diet (HFSD). HFSD, purchase from Picolab (Bethlehem, PA), was consisted of 15% anhydrous milkfat, 1.0% cholesterol, and 50% sucrose. SD or HFSD was given at least for 12 weeks up to 48 weeks and the mice were sacrificed at 12, 24, or 48 weeks after the feeding. In other groups of mice, either SD or HFSD was given for 24 weeks at which point the mice fed with HFSD were divided into two groups. For mice under HFSD, one group was fed with HFSD in combination with 20 mg/kg UDCA diluted in 0.78% Tween-80 which was administered orally through sonde for 24 weeks and the other group of mice was given 0.78% Tween-80 with HFSD. For mice under SD, 0.78% Tween-80 was also given for 24 weeks. These groups of mice were sacrificed and the liver harvested at 24 weeks after the feeding of the experimental agents with either HFSD or SD. All the mice were sacrificed at ZT8 and a portion of fresh liver tissue was fixed in 10% buffered formalin, with the remaining tissue snap-frozen in liquid nitrogen and stored at -80°C. Blood samples were collected after anesthetized by cardiac puncture, and stored at -80°C.

### Histology

Sections of liver tissue specimens, fixed in 10% formalin and embedded in paraffin wax, were stained with H&E and Sirus red for histological examination. A blinded investigator evaluated the slides for fatty change, inflammation, existence of hepatocyte ballooning, and fibrosis as described in previous studies with minor modifications [28-30]. Degree of steatosis was scored as the percentage of hepatocytes containing macrovesicular fat (grade 0: no steatosis, grade 1: <25%, grade 2: 26-50%, grade 3: 51-75%, grade 4: 76-100%). Inflammation was histologically quantified by counting inflammatory foci in 20 consecutive high-power fields (X40 objective) (average histological grade, grade 0: no foci, grade 1: <2 foci per high-power field, grade 2: ≥2 foci per high-power field). The individual scores of steatosis, inflammation and hepatocyte ballooning were added to produce an overall score, namely ‘NAFLD Activity Score’ (NAS) as previously suggested [30]. Fibrosis scores were as follows: 1, pericellular and perivenular fibrosis; 2, focal bridging fibrosis; 3, bridging fibrosis with lobular distortion; and 4, cirrhosis.

### HSC isolation and culture

HSCs were isolated from C57BL/6 male mice (16-18 weeks of age) by pronase/collagenase perfusion and density centrifugation methods using Nycodez [31,32]. Isolated HSCs were either placed and stored at -80°C in order to evaluate HSC at quiescent stage, or cultured on uncoated tissue culture dishes (1-4 × 10^4^cells/cm^2^) in DMEM supplemented with 10% FBS and penicillin in 95% air 5% CO_2_ humidified atmosphere at 37°C for culture activation. The culture activated HSCs were harvested after 7 days. HSCs were isolated from three mice for each quiescent and culture activated group.

### RNA extraction and gene expression analysis by quantitative real-time polymerase chain reaction (PCR)

Total RNA was extracted from frozen whole liver or isolated HSCs using Trizol reagent (Invitrogen, Carlsbad, CA, USA) or Qiagen mini columns (Quiagen Inc. Valencia, CA, USA) according to the manufacturer’s protocol. RNA samples were quantified by spectrophotometry. The RNA integrity was assessed using agarose gel electrophoresis and ethidium bromide staining. The RNA samples were then diluted in RNase-free water and stored at –70°C until use. Five micrograms of RNA were reverse-transcribed using RNA PCR kit version 1.2 (Takara Bio Inc, Japan) according to the manufacturer’s recommendations. Oligonucleotide primers and TagMan probe for *KLF10*, *TGFβ*, *Smad 2, 3, 7, tumor necrosis factor (TNF)α, ChREBP, sterol regulatory element binding protein (SREBP)-1c*, *collagen α1(I)* (*Col1α(I)*) and smooth muscle α-actin (αSMA) were used with 18S as internal control. The probes were obtained from Applied Biosystems (Perkin-Elmer/PE Applied Biosystems, Forster City, CA, USA), purchased as a ready-for-use form in Assays-on-Demand Gene Expression Products. The TaqMan probes were labeled at the 5’ end with the reporter dye FAM and minor groove binder (MGB) nonfluorescent quencher on the 3’end. The quantitative PCR was performed in triplicate for each sample on Step One Plus Real Time System (Applied Biosystems). Each 20-μL reaction contained 10 uL of TaqMan Fast Universal Master Mix (Applied Biosystems, Darmstadt, Germany), 1 uL of Gene Expression Mix and 2 uL of cDNA diluted in 7 μL RNase-free water. The thermal cycler conditions were 20 seconds at 95°C, 40 cycles of 5 seconds at 95°C followed by 20 seconds at 60°C. mRNA fold changes in target genes relative to the endogenous 18S control were calculated as suggested on previous studies [33].

### Protein extraction and immunoblotting

Whole liver protein lysates were extracted using a Triton-X 100 lysis buffer with protease inhibitors, and quantified using the Bradford method with BSA [34]. SDS-PAGE analysis of proteins levels was determined by means of immunoblotting using mouse anti-Klf10 (Santa Cruz, Santa Cruz, CA), and rabbit anti-β-actin conjugated to HRP (Cell Signaling Technology Inc., Trask Lane Danvers, MA). Epitope-primary antibody complexes were detected with species-specific secondary antibodies conjugated to HRP followed by ECL (Thermo Fisher Scientific Pierce, Illinois). Autoradiograms of blots were scanned and quantified using an image processor program (ImageJ from National Institute of Health). β-actin was used as a loading control.

### Measurement of liver 4-hydroxynonenal (HNE)-protein adducts in mice liver

A quantitative OxiSelect HNE-His Adduct enzyme-linked immunosorbent assay kit (Cell Biolabs, San Diego, CA, USA) was used for determination of HNE-protein adducts according to the manufacturer’s instruction. Briefly, bovine serum albumin standards or protein extracts from the liver tissues (10 μg/mL) were absorbed onto a 96-well plate overnight at 4°C. After washing 100 μL diluted anti-4-HNE-His antibody (1:1000) was added to each well and incubated for 1 hour at room temperature, followed by incubation with 100 μL of horseradish peroxidase-conjugated secondary antibody (1:1000) for an hour. After washing, 100 μL of substrate solution was added and then the reaction was stopped by adding 100 μL of stop solution after 10 minutes. The absorbance was measured at 450 nm on a microplate reader. The 4-HNE-protein adducts content in the protein samples is determined according to a standard curve prepared from predetermined 4-HNE-bovine serum albumin standards.

### Statistical analysis

All the results are presented as means ± standard error of mean (SEM). Data were analyzed by nonparametric analysis (Kruskal-Wallis or Mann-Whitney test) or one-way ANOVA with Tukey’s post hoc analysis. *P* < 0.05 was considered statistically significant. All calculations were performed with SPSS version 15.0 software (SPSS Inc., Chicago, IL, USA).

## Results

### Aging did not affect the expression of *KLF10* in the liver

**Figure 1:**
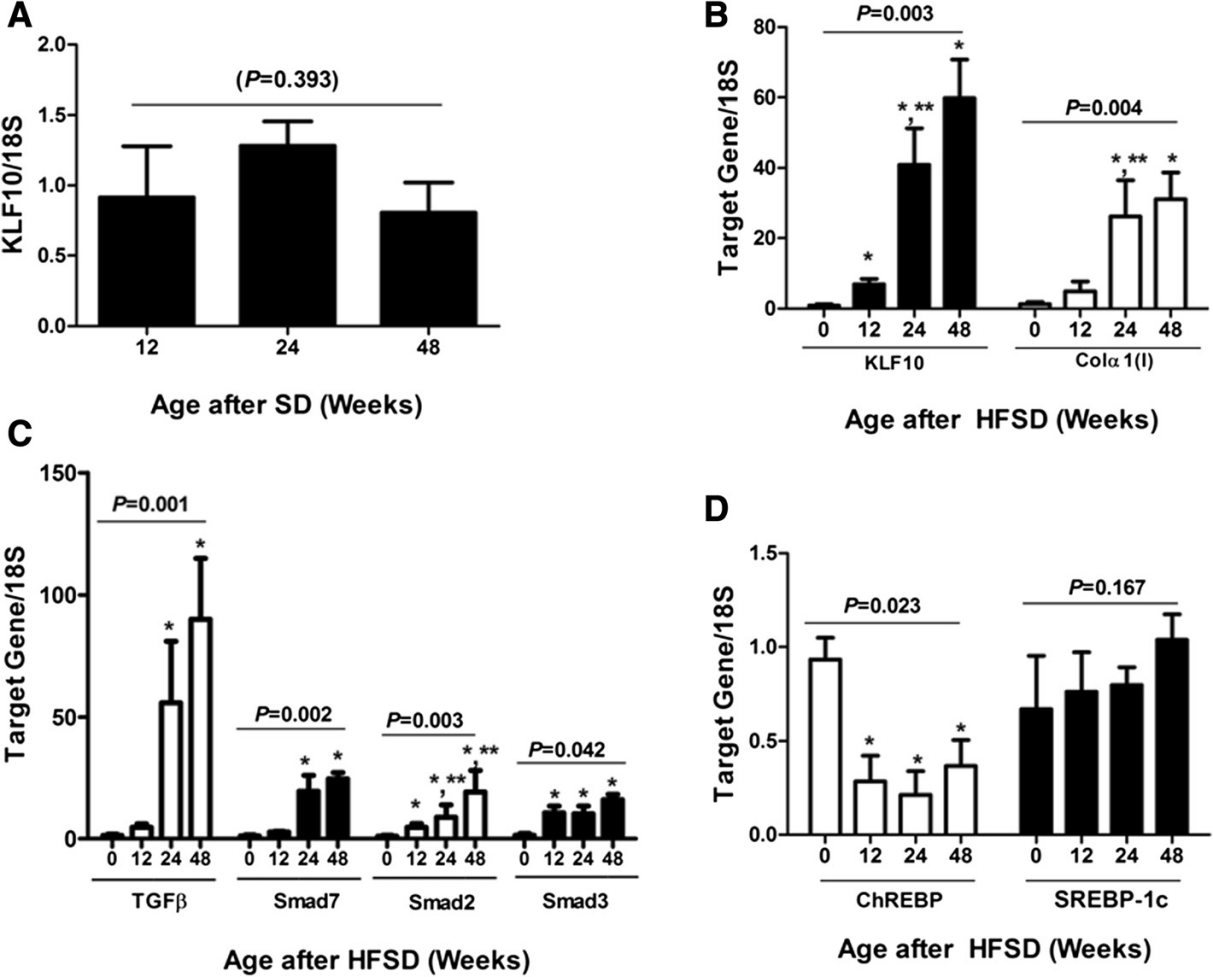
**Changes in *KLF10 *and other associated gene expression in diet induced NASH.** C57BL/6 mice were fed with high fat, sucrose diet (HFSD) and sacrificed at 12, 24, or 48 weeks after the feeding. Mice that were given standard diet (SD) for 12 weeks served as control. **A**, *KLF10* mRNA expression in the liver of SD fed mice showed no significant changes according to their age when test at the age of 12, 24, and 48 weeks after SD feeding (*P* = 0.393). **B**, Significant up-regulation of *KLF10* mRNA was observed by week 12 and thereafter when compared with the mice fed with SD which served as control (*P* = 0.003). Expression of *Colα(I)* mRNA significantly increased at week 24 and 48 (*P* = 0.004) when compared with that of mice fed with SD for 12 weeks. **C**, Significant increase of *TGFβ* mRNA was detected from week 24 and it progressed at week 48 (P = 0.001). Downstream *Smad 2*, and *3* also increased as fibrosis progressed although significant increase of *Smad 2* (*P* = 0.003) and *3* (*P* = 0.042) were demonstrated as early as week 12. Inhibitory *Smad7* increased from week 24 (*P* = 0.002). **D**, Expression of ChREBP which is reported to have a protective effect on insulin resistance, decreased with HFSD feeding, although the change was not related with either the duration of HFSD feeding or the severity of NASH (*P* = 0.023). Expression of a lipogenic gene, *SREBP-1c* showed no significant change with HFSD feeding (P = 0.167). *P < 0.05, when compared with that of mice fed with SD. **P < 0.05 when compared with that of mice fed with HFSD for the shorter duration.

Since a recent study reported that aging promoted diet induced NASH in mice [35], expression of *KLF10* mRNA in the liver was compared among the liver specimens of mice fed with SD for 12, 24 and 48 weeks (n = 8, for each ages). There was no statistically significant difference in *KLF10* expression by aging (*P* = 0.393) (Figure 1A). Expression of *TGFβ, Smad7, 2, 3, TNFα, Col1α(I)* and *ChREBP* mRNA were also tested at different age groups. These genes all demonstrated no significant changes by aging (data not shown).

### Administration of HFSD induced NASH

**Figure 2:**
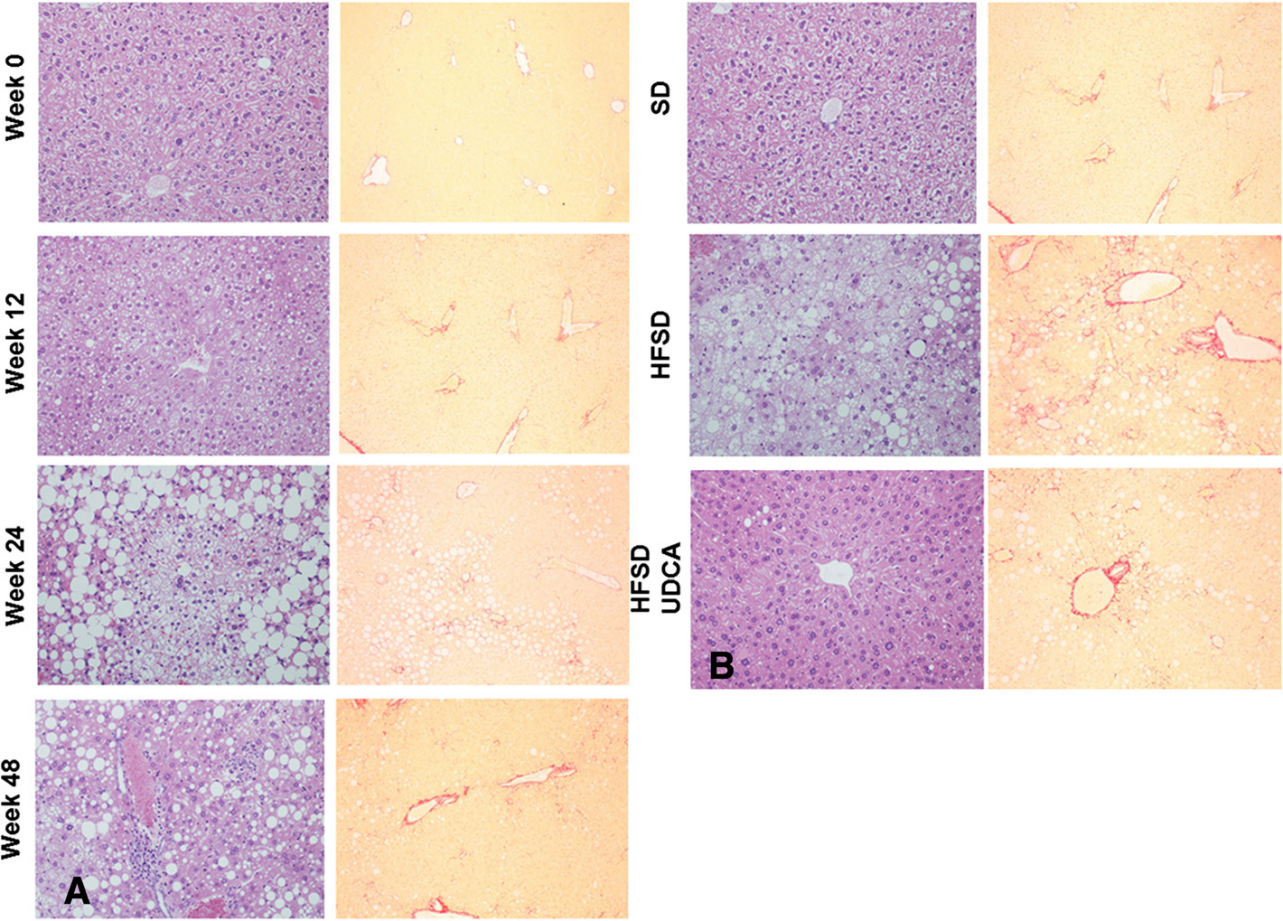
**Histological assessment of inflammation and fibrosis in diet induced NASH.** Histological analysis of the liver evaluating fatty change, inflammation, existence of hepatocyte ballooning and fibrosis was performed after H&E (X200) and Sirus red (X100) staining. **A**, Representative histological evaluation of mice fed with high fat, sucrose diet (HFSD). Liver specimens were obtained at 12, 24, or 48 weeks after the feeding. Mice fed with standard diet (SD) for 12 weeks served as the control. **B**, Representative histological assessment of mice fed with SD, HFSD and HFSD in combination with ursodeoxycholic acid (UDCA). Mice were fed with HFSD for 24 weeks and divided into two groups, from which one group was given HFSD with vehicle, and the other group was given HFSD with UDCA for another 24 weeks. Mice fed with SD and vehicle for 48 weeks served as the control.

Eight week old mice were fed with HFSD at least for 12 weeks up to 48 weeks and the mice were sacrificed at 12, 24, or 48 weeks after feeding (n = 8 for each groups) at ZT8. Mice which were given SD for 12 weeks served as control. Body weights and serum biochemical findings are summarized in Table 1. Feeding HFSD for 48 weeks resulted in increased serum cholesterol levels (P = 0.043). Giving HFSD for 24 weeks demonstrated significantly elevated serum ALT levels (P = 0.029) which worsened after 48 weeks of treatment (P = 0.000 when compared with the control; P = 0.000 when compared with that of week 24). Administration of HFSD induced liver inflammation and fibrosis, characteristics of NASH (Figure 2A). NAS was significantly increased from week 12 of HFSD feeding (P = 0.012) (Table 2). On the other hands, significant increase in fibrosis score was noticed from week 24 of HFSD feeding (P = 0.004) (Table 2).

**Table 1:** **Characterization of mice fed with HFSD**^**†**^

**Age diet**	**12 SD**^**‡ **^**(n = 8)**	**12 HFSD (n = 8)**	**24 HFSD (n = 8)**	**48 HFSD (n = 8)**
Weight	25.83 ± 0.95	30.76 ± 3.32	36.77 ± 3.15	46.8 ± 2.41*
Serum glucose (mg/dL)	125.00 ± 35.60	73.80 ± 8.11	54.33 ± 3.67	116.00 ± 18.00
Serum TG^¶^ (mg/dL)	119.00 ± 2.65	83.20 ± 14.11	74.00 ± 7.09	124.00 ± 7.23
Serum Chol^††^ (mg/dL)	82.33 ± 4.07	112.80 ± 13.62	136.67 ± 27.84	158.00 ± 5.20*
Serum ALT^‡‡^ (IU/L)	25.00 ± 3.05	81.00 ± 15.32	123.33 ± 24.25*	393.00 ± 26.58*

Data are expressed as mean ± standard of error (SE).

^†^HFSD, high fat and sucrose diet.

^‡^SD, standard chow is considered as standard diet.

^¶^TG, triglyceride.

^††^Chol, cholesterol.

^‡‡^ALT, alanine aminotransferase.

*P < 0.05, when compared with mice given SD.

Results with statistical significances are shown in bold letters.

**Table 2:** **Nonalcoholic fatty liver disease (NAFLD) activity score (NAS) and fibrosis score of mice fed with HFSD**^**†**^

**Age diet**	**12 SD**^**‡ **^**(n = 8)**	**12 HFSD (n = 8)**	**24 HFSD (n = 8)**	**48 HFSD (n = 8)**
NAS^§^	0.00 ± 0.00	3.20 ± 0.49*	7.33 ± 0.33*	6.83 ± 0.31*
Fibrosis	0.00 ± 0.00	0.02 ± 0.20	2.00 ± 0.00*	2.42 ± 0.08*^,^**

Data are expressed as mean ± standard of error (SE).

^†^HFSD, high fat and sucrose diet.

^‡^SD, standard chow is considered as standard diet

§NAS, nonalcoholic fatty liver disease activity score, which is the summation of the pathologic scores for steatosis, inflammation and hepatic ballooning.

*P < 0.05, when compared with mice given HFSD.

**P < 0.05, when compared with mice given HFSD for 24 weeks.

Results with statistical significances are shown in bold letters.

### Expression of liver *KLF10* increased in HFSD induced NASH

**Figure 3:**
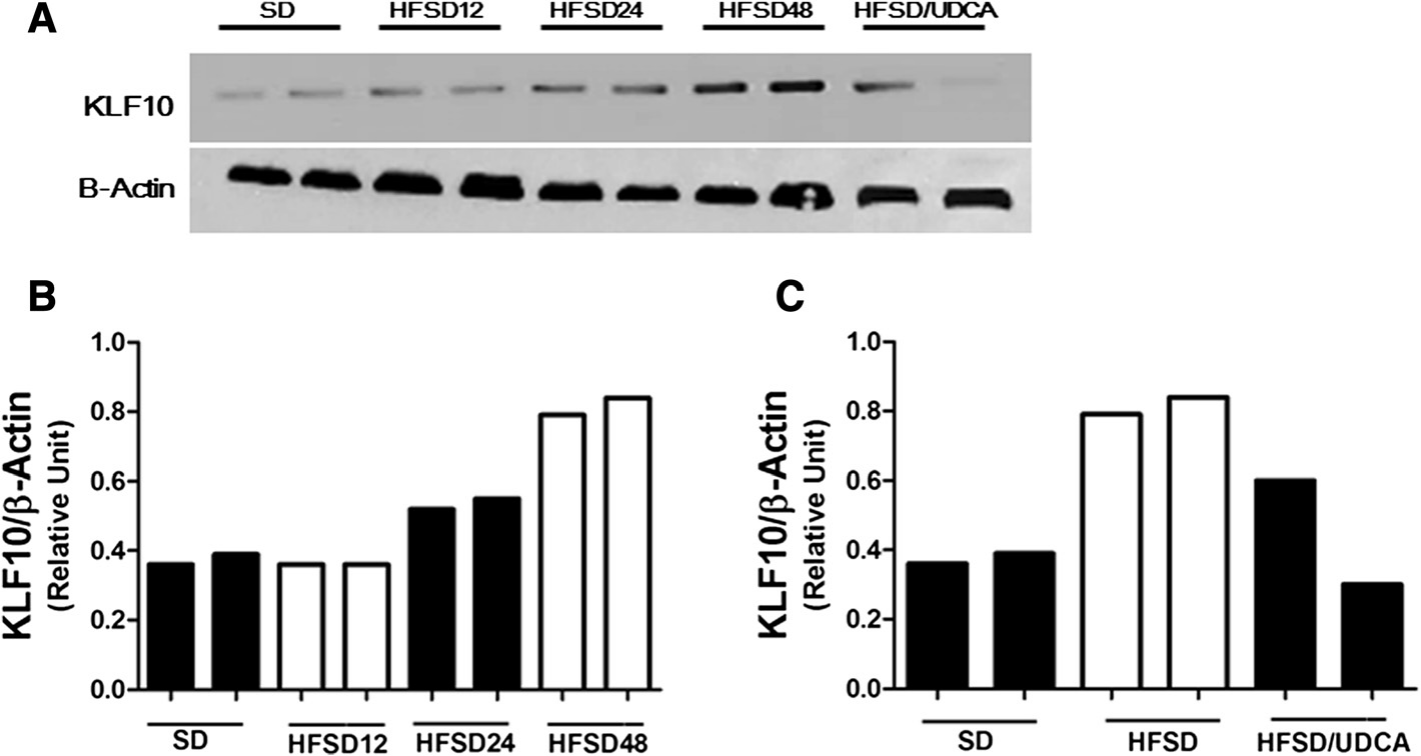
**Changes in KLF10 protein.** Western blot analysis of KLF10 was performed with β-actin antibody as a loading control. Autoradiogram of the blot was scanned and quantified using an image processor program. A representative Western blot and the result of quantitative analysis are shown. **A**, A representative Western blot autoradiogram **B**, KLF10 protein increased after 24 weeks of high fat, sucrose diet (HFSD24) feeding and more so after 48 weeks of feeding (HFSD48). Mice that were given standard diet is designated as SD. **C**, KLF10 protein which was increased after 48 weeks of HFSD feeding, was attenuated when ursodeoxycholic acid (UDCA) was given with HFSD (HFSD/UDCA).

Expression of *KLF10* in the liver significantly increased after 12 weeks of HFSD diet feeding compared with that of the mice fed with SD for 12 weeks which served as control (Figure 1B). However, *KLF10* expression at week 48 did no show significant difference when compared with that at week 24 (*P* = 0.282). Administration of HFSD also showed increased expression of KLF10 protein that was evident after 24 weeks of feeding (Figure 3A,B). In accordance with histological analysis, hepatic *Col1α(I)* mRNA expression increased by week 24 following the increase of *Klf-10* expression (Figure 1B).

### *TGFβ* mRNA increased in the liver although expression of *Smad7* was not reduced in HFSD induced NASH

Since KLF10 is initially identified as a TGFβ-inducible early gene (TIEG) and is known to act by repressing the inhibitory Smad 7 [36], we evaluated liver mRNA expression of *TGFβ, Smad7* as well as *Smad2* and *3*. Hepatic *TGFβ* mRNA expression increased from week 24 of HFSD feeding when *KLF10* mRNA expression started to increase from week 12 (Figure 1C). *Smad 2* and *3*, that are downstream mediators of TGFβ were evaluated. Although both Smad2 and 3 started to increase from week 12 of HFSD administration, Smad3 expression was not affected by the length of HFSD feeding when Smad2 expression augmented as the duration of HFSD feeding prolonged (Figure 1C). Even with the increased *KLF10* expression, inhibitory *Smad7* also increased from week 24 and thereafter (Figure 1C).

### Expression of *ChREBP* was suppressed with increase of *KLF10* expression in HFSD induced NASH

It is reported that overexpression of ChREBP induces KLF10 whereas KLF10 overexpression partially suppresses ChREBP target genes in hepatocytes [27]. We were curious how ChREBP would behave when KLF10 expression increased in HFSD induced NASH. Feeding HFSD resulted in suppressed *ChREBP* mRNA expression although the reduction rate did not correlate with the duration of HFD administration (Figure 1D). Another lipogenic gene, *SREBP-1c* was tested and demonstrated no definite difference in hepatic expression after HFSD administration (Figure 1D).

### Concurrent administration of UDCA and HFSD resulted in decreased *Colα1*(I) mRNA expression that coincided with reduction of *KLF10*

**Figure 4:**
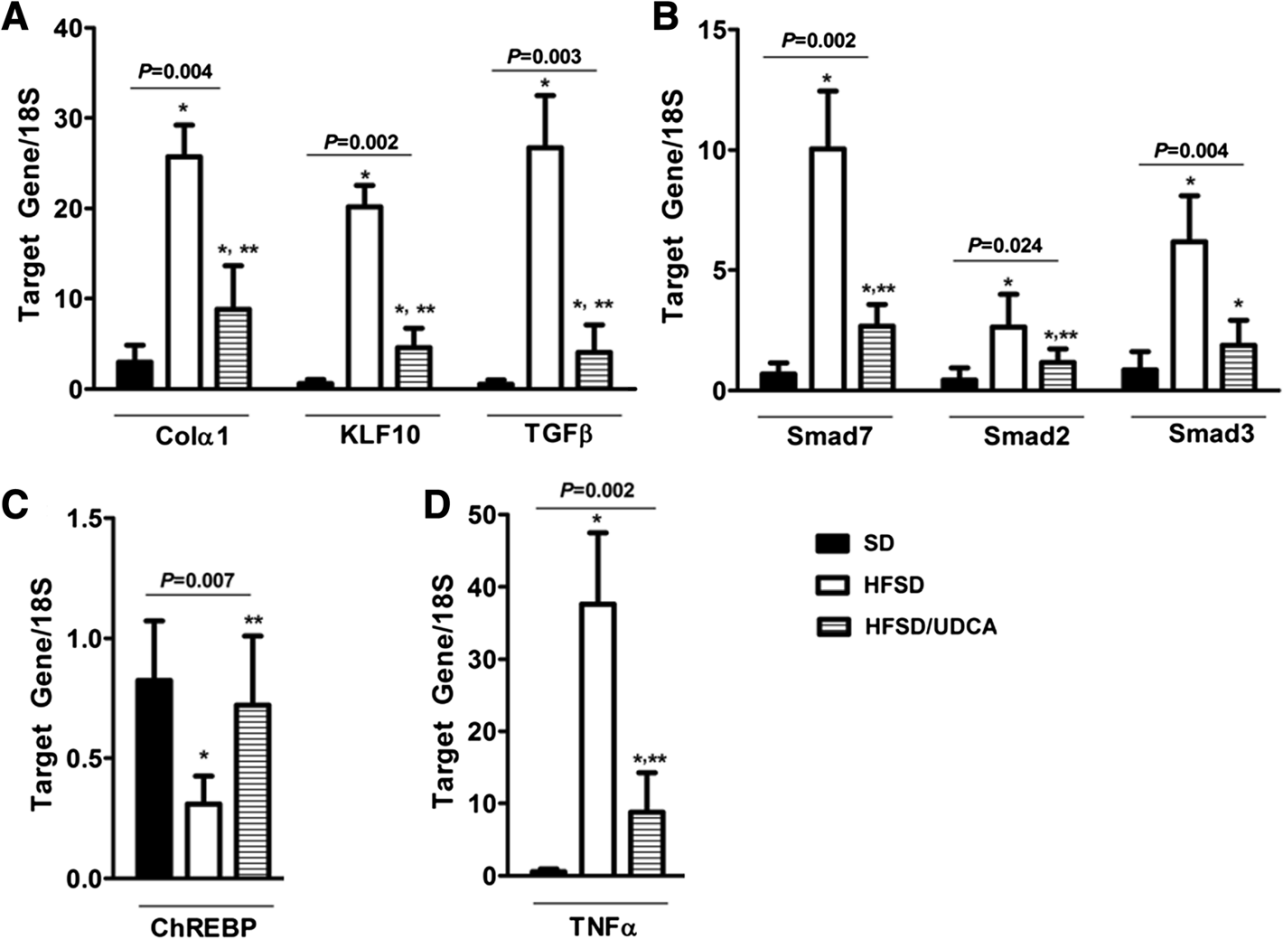
**Decreased *KLF10 *mRNA expression and improved NASH upon UDCA administration with HFSD.** C57BL/6 mice were fed with either standard diet (SD) or high fat, sucrose diet (HFSD) for 24 weeks upon which mice on HFSD were divided into two groups, and fed with either 20 mg/kg UDCA diluted in 0.78% Tween-80 or 0.78% Tween-80 only. Mice fed with SD were also given 0.78% Tween-80 as vehicle. These three groups of mice namely SD with vehicle, HFSD with vehicle and HFSD with UDCA, were fed for another 24 weeks. Total RNA was extracted from the liver. **A**, Expression of *Colα(I)* mRNA was increased by HFSD feeding but reversed when HFSD was given in combination with UDCA (*P* = 0.004). *KLF10* (*P* = 0.002) along with *TGFβ* (*P* = 0.003) mRNA expression also reduced upon UDCA administration with HFSD. **B**, TGFβ downstream signaling *Smad 2* (*P* = 0.024), and *3* (*P* = 0.004) expression significantly diminished by UDCA feeding in combination with HFSD although inhibitory Smad7 (*P* = 0.002) also reduced when UDCA was given. **C**, Expression of *ChREBP* mRNA was decreased by HFSD feeding but partially recovered when UDCA was given in combination with HFSD (*P* = 0.007). **D**, Expression of inflammatory cytokine *TNFα* mRNA which was up-regulated by HFSD feeding, reduced by UDCA administration (*P* = 0.002). *P < 0.05, when compared with that of mice fed with SD. **P < 0.05 when compared with that of mice fed with HFSD.

UDCA is reported to be beneficial in experimental NASH [37,38]. Therefore we investigated whether adding UDCA to HFSD would facilitate alleviation of NASH and be accompanied by changes in KLF10 as well as other NASH associated genes. Mice were fed with HFSD for 24 weeks and divided into two groups. They were given either HFSD with vehicle or HFSD with UDCA for another 24 weeks. Mice that were given SD received vehicle and served as control. Body weights and serum biochemical findings are summarized in Table 3. Giving HFSD resulted in increased body weight, serum cholesterol and ALT levels, and adding UDCA reversed the effect of HFSD. Pathological evaluation revealed that concurrent administration of UDCA and HFSD had decreased NAS even though improvement in fibrosis score was not evident (Table 4) (Figure 2B). Administration of UDCA with HFSD showed diminished *Col1α(I)* mRNA expression which also coincided with decreased hepatic *KLF10* and *TGFβ* mRNA (Figure 4A). Attenuated expression of KLF10 could also be detected on protein level after concurrent feeding of UDCA and HFSD (Figure 3A,C).

**Table 3:** **Characterization of mice given HFSD**^**† **^**with or without UDCA**^**‡**^

**Age diet**	**48 SD**^**§ **^**(n = 8)**	**48 HFSD (n = 8)**	**48 HFSD/UDCA (n = 8)**
Weight	35.33 ± 1.45	46.8 ± 2.41*	43.05 ± 1.42
Serum glucose (mg/dL)	366.00 ± 56.04	116.00 ± 18.00	132.67 ± 0.88
Serum TG^¶^ (mg/dL)	180.67 ± 13.96	124.00 ± 7.23	107.33 ± 28.03
Serum Chol^††^ (mg/dL)	89.33 ± 6.77	158.00 ± 5.20*	121.00 ± 16.44
Serum ALT^‡‡^ (IU/L)	70.00 ± 14.19	393.00 ± 26.58*	87.33 ± 11.57

Data are expressed as mean ± standard of error (SE).

^†^HFSD, high fat and sucrose diet.

^‡^UDCA, ursodeoxycholic acid.

^§^SD, standard chow is considered as standard diet.

^¶^TG, triglyceride.

^††^Chol, cholesterol.

^‡‡^ALT, alanine aminotransferase.

*P < 0.05, when compared with mice given SD.

Results with statistical significances are shown in bold letters.

**Table 4:** Effect of ursodeoxycholic acid (UDCA) administration on diet induced nonalcoholic fatty liver disease (NAFLD) activity score (NAS) and fibrosis score

**Age diet**	**48 SD**^**† **^**(n = 8)**	**48 HFSD**^**‡ **^**(n = 8)**	**48 HFSD/UDCA (n = 8)**
NAS^§^	0.00 ± 0.00	8.17 ± 0.40*	4.00 ± 0.44*^,^**
Fibrosis	0.00 ± 0.00	2.42 ± 0.08*	2.07 ± 0.53*

Data are expressed as mean ± standard error (SE).

^†^SD, standard chow is considered as standard diet.

^‡^HFSD, high fat and sucrose diet.

^§^NAS, nonalcoholic fatty liver disease activity score, which is the summation of the pathologic scores for steatosis, inflammation and hepatic ballooning.

*P < 0.05, when compared with mice given SD.

**P < 0.05, when compared with mice given HFSD.

Results with statistical significances are shown in bold letters.

### UDCA significantly decreased the expression of NASH associated genes

Giving UDCA with HFSD could reverse the expression of *Smad 2* and *Smad3* mRNA in the liver. However, UDCA also attenuated the expression of inhibitory *Smad7* (Figure 4B). Expression of *ChREBP* mRNA which was reduced by HFSD feeding was recovered by adding UDCA to HFSD (Figure 4C).

Expression of pro-inflammatory cytokine *TNFα* is reported to be increased in NASH [39]. UDCA administration alleviated increased *TNFα* mRNA expression (Figure 4D) in accordance with the result of histological analysis (Table 4) (Figure 1B).

### Effect of UDCA on NASH was not associated with decreased HNE protein adducts

**Figure 5:**
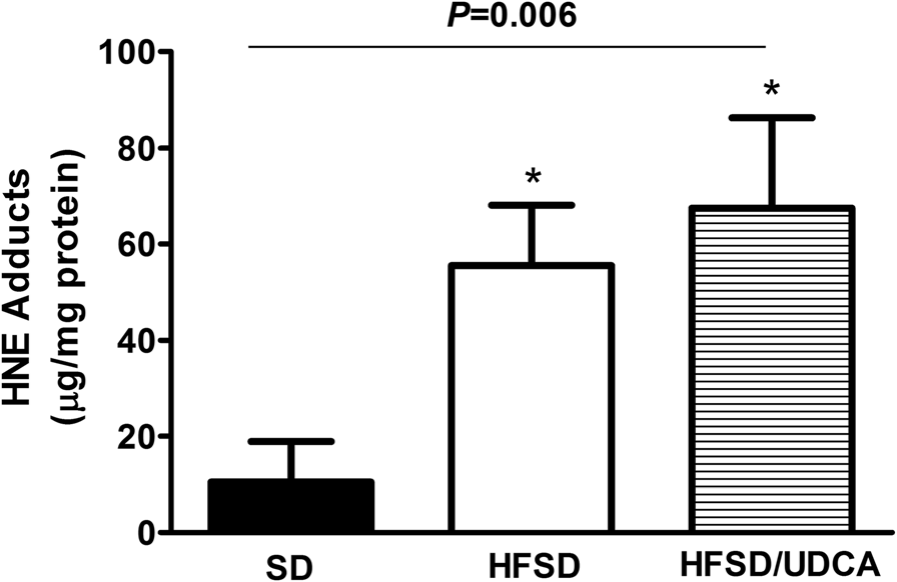
**Increased 4-hydroxynonenal (HNE)-protein adducts was not reduced by UDCA administration.** C57BL/6 mice were fed with either SD or HFSD for 24 weeks upon which mice on HFSD were divided into two groups, and fed with either 20 mg/kg UDCA diluted in 0.78% Tween-80 or 0.78% Tween-80 only. Mice fed with SD were also given 0.78% Tween-80 as vehicle. A quantitative OxiSelect HNE-His Adduct enzyme-linked immunosorbent assay kit was used for determination of HNE-protein adducts, which is the consequence of oxidative stress. Feeding HFSD significantly produced HNE adducts, and this was not reduced by UDCA administration.

Protective effect of UDCA on liver injury is frequently reported to be associated with relieving oxidative stress [38,40-42]. We evaluated whether beneficial effect of UDCA on NASH in our study was associated with decreased aldehyde-protein adduct formation, a consequence of the oxidative stress [43]. However, increased 4-HNE protein adducts by feeding HFSD was not significantly reduced by concomitant administration of UDCA (Figure 5).

### HSC activation was accompanied by increased *KLF10* expression

**Figure 6:**
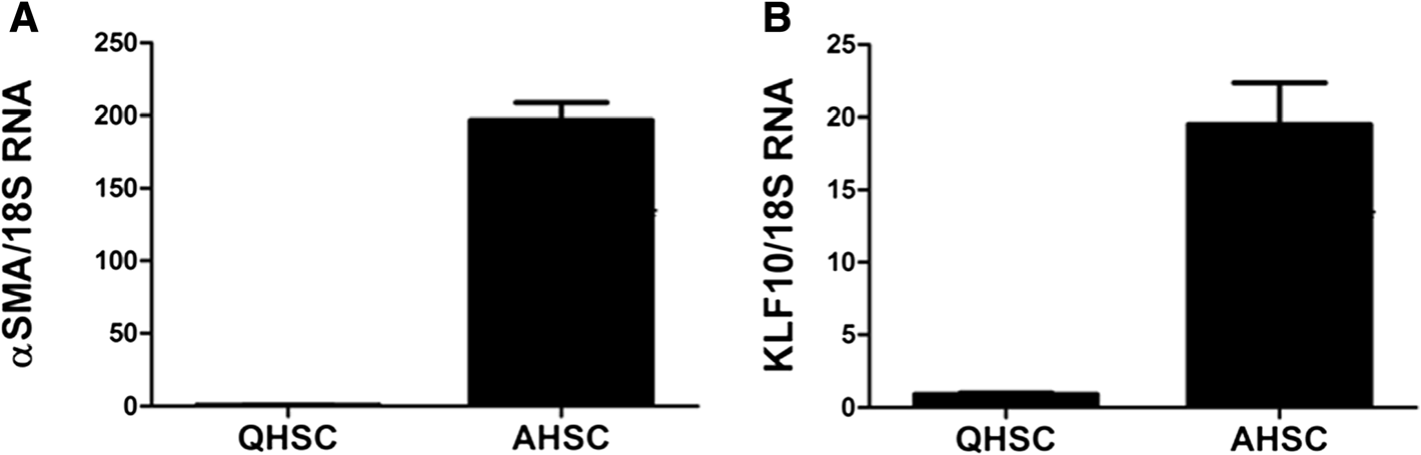
**Expression of KLF10 in activated hepatic stellate cells (HSC).** Primarily cultured HSCs were placed on a plastic dish for 7 days in order induced culture stimulated activation (AHSC). Quiescent HSCs (QHSC) without culture activation served as control. **A**, Quantification of *αSMA* mRNA, a sign of HSC activation, demonstrated the increased αSMA in activated HSCs when it was hardly detected in quiescent cells. **B**, Enhanced *KLF10* mRNA was noticed in activated HSCs when minimal expression was visible in quiescent cells.

HSCs are the major cellular component of liver fibrosis by producing ECM and collagen [44]. The profibrogenic capacity of HSCs is eminent when they are activated. Since our study demonstrated that increased KLF10 coincided with progression of liver fibrosis, we evaluated if KLF10 expression was associated with HSC activation. Primarily isolated HSCs were culture activated and expression of *KLF10* mRNA was evaluated along with αSMA, a marker for HSC activation. Primarily isolated HSCs without culture activation served as control. Expression of *αSMA* was increased in activated HSCs when it was hardly detected in quiescent cells (Figure 6A). In accordance with αSMA, expression of *KLF10* increased in activated HSCs (Figure 6B).

## Discussion

A pleiotropic cytokine TGFβ is well known for its profibrogenic effect through HSC in the liver [44]. Role of TGFβ signaling in progression of NASH associated fibrosis is also reported in several studies, making it an attractive target for the treatment [45,46]. Moreover, a study reported that TGFβ/Smad signaling is involved in regulating insulin gene and suggested its role in the development of insulin resistance [47,48]. Smad3 deleted mice are protected from insulin resistance and high fat diet induced obesity [49]. When TGFβ receptor type II is selectively deleted in hepatocytes of mice, the mice would be resistant to diet induced NASH and fibrosis [50]. Although evidences suggest that blocking TGFβ would be beneficial for NASH, targeting TGFβ at the receptor level is probably not recommendable considering the multi-functional role of TGFβ. Systemic mutation of TGF-β_1_ is found to be lethal [51]. More studies may be required in order to find targets that would alleviate the adverse effects of TGFβ with minimal influence on the critical and beneficial role of the cytokine.

KLF10 is originally named as TIEG, which would increase about 30 minutes after TGFβ treatment and rapidly return to the normal level within 2 hours [52]. Turnover of KLF10 and its subsequent role can be regulated by its proteasomal degradation, and this can modulate TGFβ/Smad-dependent transcriptional activity [53]. KLF10 suppresses cell proliferation, induce apoptosis, and modulate immune system [18,21,22,54]. Although KLF10 deficient mice are reported to grow and reproduce normally [55], these mice demonstrate defects in healing potential [56,57], and abnormally enhanced T cell function resulting in aggravated atherosclerosis [54]. A transcriptome profiling study using the liver of KLF10 deficient mice reveals that KLF10 has a significant role in regulating genes that are involved in lipid and carbohydrate metabolism [23]. ChREBP, which is also suspected to be modulated by KLF10 [27], is a transcription factor that is observed to play a protective role in insulin resistance although it would aggravate simple hepatic steatosis [26]. In our study where NASH, instead of simple steatosis without inflammation, is induced by giving HFSD, liver expression of *ChREBP* significantly decreased and its expression recovered when NASH was alleviated by UDCA administration. Depression of *ChREBP* coincided with increase of *KLF10* expression that correlated with enhanced *TGFβ* and *Col1α(I)* mRNA expression that are signs of progressive liver fibrosis. A study where *KLF10* is overexpressed in the hepatocytes demonstrates blunted ChREBP role in regulating carbohydrate metabolism [27], and it can be speculated that increased *KLF10* along with attenuated *ChREBP* expression may have some association with progression of NASH.

In addition to liver fibrosis, it is well established that inflammation is also very important in development and progression of NASH [30,39]. Previous reports suggest the important role of KLF10 as a modulator of regulatory T cell function and deleting KLF10 demonstrates enhanced inflammatory reaction in cardiovascular system [54]. Although it is observed that high fat diet induced steatosis is accompanied by depletion of hepatic regulatory T cells [58], association of this regulatory T cell depletion and KLF10 has never been investigated. In our study, *KLF10* up-regulation was coincided with increase in *TNFα* and worsening of histologically evaluated NAS, and the precise mechanistic role of KLF10 in inflammatory reaction of NASH should be sought in the future, possibly through a KLF10 deletion study.

As stated earlier, KLF10 is known to be a TGFβ induced early gene which would rapidly return to the normal level within 2 hours after the stimulation. However, in the setting of HFSD induced NASH or culture activated HSCs, the insults are given repeatedly and these continuous stimulations may have resulted in chronic activation of KLF 10.

Although clinical trials fail to show a meaningful effect of UDCA on alleviating NASH induced fibrosis, beneficial role of UDCA has been supported by several animal studies. These discrepancies may be explained by high UDCA dose used in animal studies that could hardly be achieved in humans [39]. Nevertheless, since the goal of our study was to investigate whether improvement in NASH would be accompanied by modulation of KLF10 expression, UDCA was used as a safe means of investigation. We observed that concurrent administration of UDCA and HFSD reduced *Col1α(I)* mRNA production. This improvement was accompanied by decreased *KLF10* and *TGFβ* as well as recovered *ChREBP* expression in the liver, suggesting that enhanced *KLF10* transcription might be related with NASH progression although the precise cause-and-effect mechanism should be delineated by another interventional study.

Our study revealed that activated HSCs were accompanied by increased *KLF10* when *KLF10* expression was negligible in quiescent HSCs. This finding still cannot explain whether KLF10 facilitates the progression of NASH fibrosis or the consequence of the liver fibrosis. However, it is worth noting that KLF10 expression is enhanced in ECM producing HSC.

The beneficial effects of UDCA on NASH can be explained by its anti-inflammatory, hepatoprotective effect as well as its role as an antioxidant [38,41,59,60]. Although our study showed significantly decreased *TNFα* expression after UDCA administration, it failed to show meaningful decrease in 4-HNE protein adducts, which is the consequence of oxidative insult. However, oxidative stress can be evaluated by multiple means and not having decreased 4-HNE protein adducts production cannot conclusively deny UDCA’s role as an antioxidant in relieving NASH. Our study rather suggests that UDCA takes through multiple pathways in alleviating NASH.

## Conclusions

To our knowledge, this is the first study that evaluated the expression of KLF10 in NASH and HSCs. Our study demonstrated that *KLF10* expression was significantly increased in diet induced NASH and ECM producing activated HSCs. We also observed that this up-regulation of *KLF10* was accompanied by increased TGFβ signaling genes and suppressed *ChREBP* expression which was known to improve insulin resistance. These observations may suggest possible association of KLF10 in progression of NASH. Further studies should be warranted to assess the specific role and mechanism of KLF10 in NASH associated liver fibrosis.

## Abbreviations

NAFLD: Nonalcoholic fatty liver disease; NASH: Nonalcoholic steatohepatitis; TGFβ: Transforming growth factor β; KLF10: Kruppel-like-factor 10; TNFα: Tumor necrosis factor α; ChREBP: Carbohydrate response element-binding protein; SREBP-1c: Sterol regulatory element binding protein 1c; Col1α(I): Collagen α1(I); αSMA: Smooth mucle α-actin; UDCA: Ursodeoxycholic acid; ECM: Extracellular matrix; ZT: Zeitgeber time; HSC: Hepatic stellate cell; HFSD: High fat and sucrose diet; SD: Standard diet; NAS: Nonalcoholic fatty liver disease activity score; TIEG: TGFβ inducible early gene; HNE: Hydroxynonenal; SEM: Standard of mean.

## Competing interests

The authors declare that they have no competing interest.

## Authors’ contributions

JK: study concept and design, data acquisition, drafting of manuscript. KSL: study concept and design, project coordination, data analysis, writing manuscript. HYC: processing of the experiment, data analysis. WKL: study concept, processing of the experiment especially regarding KLF10. JIL: obtainment of grant, study concept and design, analysis and interpretation of data, drafting of manuscript. All authors read and approved the final manuscript.
